# Primary care management for optimized antithrombotic treatment [PICANT]: study protocol for a cluster-randomized controlled trial

**DOI:** 10.1186/1748-5908-7-79

**Published:** 2012-08-28

**Authors:** Andrea Siebenhofer, Lisa R Ulrich, Karola Mergenthal, Ina Roehl, Sandra Rauck, Andrea Berghold, Sebastian Harder, Ferdinand M Gerlach, Juliana J Petersen

**Affiliations:** 1 Institute of General Practice, Goethe-University Frankfurt am Main, Frankfurt, Germany; 2 Institute for Medical Informatics, Statistics and Documentation, Medical University of Graz, Graz, Austria; 3 Institute of Clinical Pharmacology, Center for Drug Research, Development and Safety, Goethe-University Frankfurt am Main, Frankfurt, Germany

**Keywords:** Oral anticoagulation, Best-practice model, Case management

## Abstract

**Background:**

Antithrombotic treatment is a continuous therapy that is often performed in general practice and requires careful safety management. The aim of this study is to investigate whether a best-practice model that applies major elements of case management and patient education, can improve antithrombotic management in primary healthcare in terms of reducing major thromboembolic and bleeding events.

**Methods:**

This 24-month cluster-randomized trial will be performed with 690 adult patients from 46 practices. The trial intervention will be a complex intervention involving general practitioners, healthcare assistants, and patients with an indication for oral anticoagulation. To assess adherence to medication and symptoms in patients, as well as to detect complications early, healthcare assistants will be trained in case management and will use the Coagulation-Monitoring List (Co-MoL) to regularly monitor patients. Patients will receive information (leaflets and a video), treatment monitoring via the Co-MoL and be motivated to perform self-management. Patients in the control group will continue to receive treatment as usual from their general practitioners. The primary endpoint is the combined endpoint of all thromboembolic events requiring hospitalization and all major bleeding complications. Secondary endpoints are mortality, hospitalization, strokes, major bleeding and thromboembolic complications, severe treatment interactions, the number of adverse events, quality of anticoagulation, health-related quality of life, and costs. Further secondary objectives will be investigated to explain the mechanism by which the intervention is effective: patients’ assessment of chronic illness care, self-reported adherence to medication, general practitioners’ and healthcare assistants’ knowledge, and patients’ knowledge and satisfaction with shared decision making.

Practice recruitment is expected to take place between July and December 2012. Recruitment of eligible patients will start in July 2012. Assessment will occur at three time points: baseline and follow-up after 12 months and after 24 months.

**Discussion:**

The efficacy and effectiveness of individual elements of the intervention, such as antithrombotic interventions, self-management concepts in orally anticoagulated patients, and the methodological tool of case management, have already been extensively demonstrated. This project foresees the combination of several proven instruments, as a result of which we expect to profit from a reduction in the major complications associated with antithrombotic treatment.

**Trial registration:**

Current Controlled Trials ISRCTN41847489

## Background

Oral anticoagulation has been shown to be highly effective in preventing thromboembolic complications in patients for whom it is indicated [1,2]. Atrial fibrillation, the incidence of which increases with age and approaches 10% for individuals aged ≥80 years, carries the major risk of a stroke [3,4]. In Germany, large-scale epidemiological data are unavailable, hence it can only be estimated that about 1 million people are suffering from atrial fibrillation. The German Competence NETwork on Atrial Fibrillation (AFNET) has initiated a large nationwide patient registry to evaluate the current daily care of patients with atrial fibrillation in Germany [5]. The registry shows that anticoagulation for stroke prevention was given to 71% of patients considered eligible according to applicable guidelines and to 48.4% of low-risk patients, for whom guidelines do not recommend anticoagulation, meaning that overtreatment may also exist. In an international study that also included German patients, it was reported that only about 65% of patients requiring treatment and not presenting any contra-indications received anticoagulation treatment [6]. In addition, due to relatively narrow therapeutic ranges, it is often only a small percentage of international normalized ratio (INR) values that are found to be within the target range. This proportion, as German studies of patients receiving routine care have shown, can be as low as <40% of INR measurements [7,8]. As it is well known that about 44% of bleedings occur when INRs are above the therapeutic range, that about 50% of thromboembolic events happen when INR values are too low [9], and that improved oral anticoagulation is an indirect parameter for the reduced incidence of thromboembolic and hemorrhagic events, there is a real need to improve anticoagulation control. Furthermore, our own analysis of the oral anticoagulation therapy treatment process in 16 general practices in Germany identified safety gaps. These were mainly found in documentation, patient participation, knowledge of side effects, and interactions [10].

The new oral agents dabigatran, a direct thrombin inhibitor, and rivaroxaban, a direct Factor Xa inhibitor, have recently been approved for patients with a long-term indication for antithrombotic treatment. Dabigatran has been approved for patients with nonvalvular atrial fibrillation and one additional risk factor for stroke, with approval primarily based on the results of the RE-LY study [11]. Compared to warfarin, dabigatran administered at a dose of 150 mg was associated with lower rates of stroke and systemic embolism but similar rates of major bleedings. However, in November, the European Medicines Agency (EMA) updated the safety label for dabigatran after reports of several cases of fatal bleeding events in patients of relatively high age that had renal insufficiency and low body weight [12]. Rivaroxaban was approved in December 2011 for two long-term indications (patients with nonvalvular atrial fibrillation and one additional risk factor for stroke and to prevent the reoccurrence of deep venous thrombosis and pulmonary embolism). With regard to atrial fibrilliation, data primarily based on the ROCKET-AF study could not show superiority versus warfarin in terms of a reduction in the incidence of strokes, systemic embolisms, or major bleeding complications [13]. However, as both new oral agents have recently been approved, special caution relating to drug interactions, renal insufficiency, and low body weight (as with dabigatran) is required because unknown risks may exist in an unselected patient population [14].

The efficacy and effectiveness of antithrombotic treatment have already been extensively demonstrated. However, no study has yet analyzed a complex intervention that incorporates further elements of proven benefit, such as self-management of OAC patients and case management in general practices. Systematic reviews [15-17] and a randomized controlled trial (RCT) in elderly patients [18] have confirmed that thromboembolic events and deaths can be reduced in patients performing self-management, with no difference in the incidence of major hemorrhagic complications. In the long-term RCT of elderly patients, self-management of OAC was able to improve general treatment satisfaction, as well as having a positive effect on treatment-related quality of life [19]. Case management is generally defined as a clinical, educative, and social service provided to individuals with high needs [20]. It mostly includes planned services for coordinating care, systematic follow-up, and evidence-based guidelines. Beneficial effects of case management in primary care have been shown—partially based on our own results—for many chronic diseases (*e.g.*, diabetes [21], depression [22,23], chronic heart failure, osteoarthritis [24] and other diseases, as well as continuous therapies [25]). As it is proactive and patient oriented, case management can be expected to increase adherence to antithrombotic therapy and, therefore, improve quality of care.

The aim of this study is, therefore, to improve antithrombotic management in primary healthcare by applying major elements of case management, including patient education, and to test their effectiveness in terms of reducing major thromboembolic and bleeding events.

## Methods/design

### Study design

**Figure 1:**
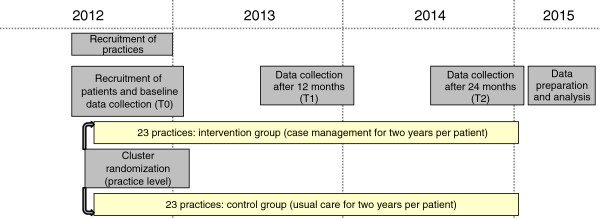
Description of schedule and expected duration of the PICANT trial.

Primary care management for optimized antithrombotic treatment (PICANT) is to be a cluster RCT with the general practice as the unit of randomization (see Figure 1). Since healthcare assistants (HCAs) and general practitioners (GPs) trained in the intervention will not be in a position to provide care as usual, a clustered design (with practices as clusters of 15 patients per practice) was chosen in order to reduce treatment group contamination. Practices will be randomly allocated to the case management or usual care arm in a ratio of 1:1. Practice allocation to treatment groups will be performed using the web-based randomization tool “Randomizer for Clinical Trials,” developed by the Institute for Medical Informatics, Statistics and Documentation, Medical University of Graz (http://www.randomizer.at). After patient recruitment and baseline assessment have been completed in a practice, a member of the Institute of General Practice will use the web-based randomization tool to obtain information on the allocated treatment group. Allocation concealment will take place after completion of the baseline documentation for all study patients from a specific practice, but before the intervention begins.

### Practice and patient recruitment

The trial will be primarily conducted in general practices in the state of Hesse, Germany.

Practices are eligible for the trial if they provide health services to persons with German statutory health insurance and have a software system at their disposal that is capable of detecting potentially eligible patients.

Practices already participating in studies aimed at improving quality of oral anticoagulation will be excluded. Participating GPs must sign an informed consent form and agree to implement the study protocol. Potentially eligible practices will be identified by means of an address database provided by the Association of Statutory Health Insurance Physicians of Hesse. The practices will be stratified according to the number of inhabitants of the postal area in which the practice is located. Based on our own recruitment experience during previous studies involving general practices in Hesse [26], we would expect to have to contact >500 practices to recruit the required number of practices for the trial. In a first step, we will write a formal letter inviting approximately 500 randomly selected practices to participate in the study. If the required number of participating practices is not achieved, further randomly selected practices (in steps of 250 practices at a time) will be contacted and asked to participate. The random samples will be selected by means of the random number generator function in Microsoft Excel® (Microsoft Corporation, Redmond, WA, USA). The random selection of practices will be performed by a member of the study team in the presence of a third person who is not involved in the study.

Each participating practice will be asked to create a screening list of potentially eligible patients by means of their software and on the basis of predefined instructions. At the first practice visit, the GP will go through the screening list in the presence of a member of the study team and assess the inclusion and exclusion criteria of the patients until 30 eligible patients have been identified. To avoid a selection bias, the order of the patients assessed for eligibility will be randomly chosen by means of the random number generator function in Microsoft Excel. The 30 eligible patients will then receive a written invitation from the GP to participate in the study. Once 15 patients have been included, patient recruitment will be ceased. This recruitment strategy was developed in accordance with previous studies [26,27]. The process used to identify eligible study patients was pilot tested in seven practices and found to be feasible.

### Inclusion and exclusion criteria for patients

Adult patients of ≥18 years of age and with a long-term indication for oral anticoagulation (atrial fibrillation/ flutter, recurrent venous thromboembolism or pulmonary embolism, mechanical heart prosthesis, and others, such as hereditary coagulopathy, intracardial thrombosis) and an indication for coumarins, antiplatelet therapies, or the new antithrombotic agents rivaroxaban and dabigatran will be included in the study. They should regularly attend the GP’s practice and must sign an informed consent form. Exclusion criteria for patients are dementia, diseases resulting in a life expectancy of less than six months, psychosis, severe sight disorders or auditory defects, alcohol or drug abuse, residence in institutions (*e.g.*, nursing homes or residential care homes) that do not allow study participation, and a lack of German language skills.

### Outcome measures

The primary patient-relevant endpoint of the trial is the combined endpoint of all thromboembolic events requiring hospitalization and all major bleeding complications, as assessed by the GP and documented in the case report form. Major bleeds include (1) fatal bleeding; (2) symptomatic bleeding in a critical area or organ, such as intracranial, intraspinal, intraocular, retroperitoneal, intraarticular or pericardial, or intramuscular with compartment syndrome; and/or (3) bleeding causing a fall in the hemoglobin level of 20 g/L (1.24 mmol L-1) or more or leading to the transfusion of two units of packed red blood cells [28]. Thromboembolic events requiring hospitalization include arterial embolism (stroke, transient ischemic attack, myocardial infarction, peripheral arterial embolism), venous thromboembolism (*e.g.*, deep venous thrombosis, pulmonary embolism), and valve thrombosis [18,29].

The following key secondary endpoint(s) will be evaluated by means of the case report forms (filled in by the GPs) and patient questionnaires: all-cause and cause-related mortality rates, frequency and duration of hospitalization, number of recurrent strokes (ischemic and hemorrhagic stroke), major bleeding and thromboembolic complications (as defined for the primary endpoint), the number of patients with at least one potentially severe treatment interaction, total number of potentially severe treatment interactions involving oral anticoagulants, the number of adverse events, quality of anticoagulation (*e.g.*, time within therapeutic range) [30], health-related quality of life (EQ-5D) [31], and costs.

Further secondary objectives will be investigated to explain the mechanism by which the intervention is effective: patients’ assessment of chronic illness care (PACIC) [32], self-reported adherence to medication (Morisky Questionnaire) [33], general practitioners’ and healthcare assistants’ knowledge (self-developed knowledge questionnaire), patients’ knowledge (questionnaire developed by Hua) [34], and satisfaction with shared decision making (Man-Song Hing test) [35].

All primary and secondary outcomes will be measured at patient level at baseline (T0) and at follow-ups at 12 months after baseline (T1) and 24 months after baseline (T2). All events will be documented in case report forms and cross-checked by reviewing doctors’ reports, discharge letters, and death certificates. Allocation concealment will take place after completion of the baseline documentation for all study patients from a specific practice, but before the intervention begins.We will conduct qualitative interviews with patients, HCAs, and GPs to evaluate the perceptions of and satisfaction with practice-based case management and to study the general practitioners’ reasons for changing medication to the new antithrombotic drugs (dabigatran or rivaroxaban).

### Intervention in the treatment groups

At the first practice visit, all practices (before randomization) will be provided with the current evidence-based guideline for GPs, “Anticoagulation” of the Guideline Group of Hesse, as a “recommended standard” [36] and a standardized information leaflet (issued by the German Society of General Practice and Family Medicine) for their patients [37].

### Intervention group

Practices randomized to the intervention group will receive a complex intervention, including the provision of additional tools and guidelines to GPs and their practice teams. The HCAs will be trained in performing case management and educating patients (including a patient information leaflet and a video developed by Hua *et al.*) [34]. To assess adherence to medication and symptoms in patients, as well as detect complications early, HCAs will be trained in case management and will regularly monitor patients by means of the Coagulation-Monitoring List (Co-MoL). Practice routines will be improved. In addition, the GPs will be contacted immediately after randomization and provided with further explanations of case management by telephone. Quality circles will be held three times during the course of the trial (shortly after baseline assessment and after 12 and 24 months) to discuss the practical problems of anticoagulation and individual case reports.

For patients, the intervention will include information (patient information leaflet and a video developed by Hua *et al.*[34]), treatment monitoring via the Co-MoL, and encouragement to participate in a self-management course where they will learn how to carry out self-testing and self-dosing.

### Training of the healthcare assistant

At an interactive one-day workshop, one HCA from each practice assigned to the intervention group will be trained to perform case management and educate patients. The main elements of the session will be to inform patients about their disease and treatment conditions, to encourage patients to perform self-management of oral anticoagulation if they are taking coumarins, and to monitor symptoms and their adherence to antithrombotic treatment. HCAs will also be trained to detect complications early and to assess adverse events, such as major or minor thromboembolisms or bleeding complications, as well as drug-related side effects and interactions. The Co-MoL will be extensively explained. Training in educating patients willing to perform self-management will be adapted from the teaching course developed by Sawicki *et al.*[8]. For those HCAs who are not willing to give self-management courses in their practices, a list of teaching centers where patients can be transferred will be provided. A model for a similar course was successfully developed as part of our Primary Care Monitoring for Depressive Patients Trial (PRoMPT) study [22].

### Control group

For the duration of the trial, the patients in the control group will continue to receive treatment as usual from their GP.

### Pilot testing

Between November 2011 and March 2012, pilot tests were conducted in seven practices in order to try out the process for the identification of eligible patients, as well as to check the comprehensibility of the instruments. The piloting led to an adaptation of the processes and questionnaires. The results of the piloting will, however, not be included in the final analyses.

### Sample size calculation

Sample size was calculated by using the primary combined endpoint of all thromboembolic events requiring hospitalization and all major bleeding complications in the 24-month intervention period.

Based on data from previously published studies [11,18,38], we anticipate an event rate of 15% over the 24-month period in the routine-care group. A 50% reduction in thromboembolic events and major bleedings is expected in the case-management group. To detect a 50% reduction between the two treatment groups using a chi-square test with a two-sided significance level of 5% and a power of 80%, a sample size of 278 patients per group is needed. To account for the design effect in this cluster RCT, the sample size will have to be inflated by the design effect (DE), where DE = 1 + (*m* − 1) ICC (*m* is the average cluster size and ICC is the intraclass correlation coefficient). In accordance with Campbell *et al.*, 2007, and Campbell 2000 we will use an estimate of 0.01 for the ICC [39,40]. On the assumption that it will be possible to identify about 30 eligible patients in each general practice and that 50% of these can be recruited for the study, the adjusted sample size per group is 317. To account for drop outs and incomplete follow-up, our objective is to include 345 patients (23 general practices) per group. In addition, we will include a further four practices in each group in order to be prepared for any practice loss. The sample size calculation was performed using nQuery Advisor 7.0 (Statistical Solutions Ltd., Cork, Ireland).

### Statistical hypotheses, methods, and analyses

The primary analysis of all target criteria will be performed for all randomized practices and participating patients in accordance with the intention-to-treat (ITT) principle. The first event that occurs in a patient will be used for analysis. A modified ITT analysis with patients switching to the new antithrombotic treatment (rivaroxaban and dabigatran), censored at the time of switching, will be performed if it is applicable. The primary endpoint will be analyzed using an extended Cox proportional hazards model to take the effects of clustering into account. A *p* value of less than 5% will be considered significant. To assess bias due to missing data, we will perform sensitivity analyses. Detailed exploratory analysis of all secondary endpoints will be conducted. Subgroup analyses for gender are planned. The statistician will be blinded with regard to treatment allocation. For statistical analysis, SAS 9.2 (SAS Institute, Inc., Cary, NC, USA) and SPSS version 19 (IBM, Armonk, NY, USA) will be used. A detailed description of the statistical methods used in this study will be provided in a statistical analysis plan (SAP), which will be finished before database lock.

### Ethics and legal aspects

The project will be carried out in conformation with the Medical Association’s code of conduct and good clinical practice (GCP) in line with the World Medical Association Declaration of Helsinki [41]. The trial was checked and approved prior to the start of the study by the ethics committee of Frankfurt University Hospital (ethics committee approval obtained on June 26^th^, 2011). The study is registered at http://www.controlled-trials.com (ISRCTN41847489).

### Timeframe of the study

Practice recruitment is expected to take place between July 2012 and December 2012 (see Figure 1). Recruitment of eligible patients and baseline data collection will start in July 2012. After cluster randomization, teams in intervention practices will be consecutively trained between August and December 2012, prior to the beginning of the intervention. Data collection for the 12-month assessment is scheduled from June 2013 to January 2014 and from June 2014 to January 2015 for the 24-month assessment.

## Discussion

Antithrombotic treatment is a continuous therapy that is often performed in general practice. Therapy with coumarins, which have a narrow therapeutic range, bears a considerable risk of adverse thromboembolic and bleeding events, particularly in the case of dose deviations when INR values are outside the target range. Patients on new antithrombotic treatments—who will be included in the study as well—also require careful safety management, and this will continue to be a challenging task in certain risk populations [14]. According to the Institute for Safe Medication Practices, coumarins are one of the five highest-risk medications that are in wide use, and their utilization can lead to severe or even lethal adverse events [42].

This cluster RCT compares a best-practice model involving HCAs trained to provide case management with usual care under “real-world conditions.” HCAs have increasingly been recognized as an underexploited resource in chronic care. The model is a complex intervention, but the effectiveness of all major components has been proven. Only the reliable and safe management of antithrombotic treatment will increase its use as it would result in increased treatment certainty among treating doctors and beneficial consequences in the population at risk. Immediate consequences in terms of risk reduction (particularly of stroke prevention) will occur, if the case-management components of the program are as effective as expected.

Case management by HCAs will require more resources within the general practice compared to usual care, but this will likely be offset by the overall benefits. In addition to the medical effects in terms of benefits and harm, economic effects will also be evaluated and resource consumption will be documented and monitored (risk–benefit assessment).

## Competing interests

The authors declare that they have no competing interests.

## Authors’ contributions

AS, JJP, LRU, IR, KM, SR, AB, and SH developed the intervention and study protocol. FMG contributed to the development of the study protocol. AS and JJP wrote the first draft of the manuscript. All authors critically revised it. All authors read and approved the final manuscript.
